# Irrelevant background context decreases mnemonic discrimination and increases false memory

**DOI:** 10.1038/s41598-021-85627-2

**Published:** 2021-03-18

**Authors:** Mihály Racsmány, Dorottya Bencze, Péter Pajkossy, Ágnes Szőllősi, Miklós Marián

**Affiliations:** 1 Department of Cognitive Science, Budapest University of Technology and Economics, Egry Jozsef utca 1, 1111 Budapest, Hungary; 2 Institute of Cognitive Neuroscience and Psychology, Research Centre for Natural Sciences, Budapest, Hungary

**Keywords:** Human behaviour, Neuroscience

## Abstract

One of the greatest commonplaces in memory research is that context improves recall and enhances or leaves recognition intact. Here we present results which draw attention to the fact that the reappearance of irrelevant and unattended background contexts of encoding significantly impairs memory discrimination functions. This manuscript presents the results of two experiments in which participants made indoor/outdoor judgements for a large number of object images presented together with individual, irrelevant and presumably unattended background scenes. On a subsequent unexpected recognition test participants saw the incidentally encoded target objects, visually similar lures or new foil objects on the same or new background scenes. Our results showed that although the reappearance of the background scene raised the hit rate for target objects, it decreased mnemonic discrimination, a behavioral score for pattern separation, a hippocampal function that is affected in early dementia. Furthermore, the presence of the encoded background scene at the recognition test increased the false recognition of lure objects, even when participants were explicitly instructed to neglect the context scene. Altogether these results gave evidence that if context increases recognition hits for target memories, it does so at the cost of increasing false recognition and diminished discriminability for similar information.

## Introduction

### Positive mnemonic effects of context

It has been known for centuries that the presence of the learning event’s context at the moment of recollection greatly improves recall performance^1–4^. However, the scientific results regarding the effect of context on recognition are far from clear^4,5^. A range of experimental studies found no or weak contextual effects in face recognition experiments^6–8^. Context appeared to have an effect on recognition tests only if the encoding was intentional and/or the context changed the meaning of the studied information. In the light of these results, Baddeley^1^ suggested to discriminate between independent and interactive contexts. The latter one changes the meaning of the to-be-recognized information, while independent context is assumingly stored together with the stimulus with no change. In this case, context affects recall performance because the contextual features are utilized as extra cues during retrieval. However, according to Baddeley^1^, the presence of independent contextual information only helps recall, and not recognition. Contrary to this view, a meta-analysis of 75 experiments identified non-associative processing during study as a key factor for beneficial environmental context effects on recognition^4^. Furthermore, analyzing the effect of environmental context, the authors found that inter-item associative processing pronouncedly decreased the effect size, whereas the combined manipulation of the place and experimenter markedly increased the effect size. They also revealed a large effect size with a retention interval of one day or more, and most interestingly, there were no differences in the effect sizes between recall and recognition. It is important to emphasize that the experiments reporting context effects in recognition memory decisions used mainly intentional learning paradigms^5^. In these situations, the subjects know that they need to memorize the information they have just seen or heard during learning, so the contextual information can be used as a cue already in the encoding phase and later in the recognition phase. In this case, the context is no longer an irrelevant environmental background information, but serves as a recall cue to help remember.

In one of the rare examples of studies using incidental learning, Hayes et al.^9^ examined the effect of scene contexts on object-related recognition memory performance. In a series of experiments, they presented unique objects either embedded in a visually rich scene or on a white background. The context change in this case meant the lack of context during the test. That is, during the study, the object was presented on a scene, whereas during the test, it was shown on a white background. Using both incidental and intentional learning instructions, the authors found decreased recognition performance when the context was changed between encoding and a yes/no recognition memory task. Importantly, the context change-dependent decrease in hit rate was a relative decrease compared to the condition where the subject was presented with a white background during both learning and testing. However, in the study of Hayes and colleagues^9^ no highly similar lure discrimination was required, i.e., only correct and false recognition rates were measured. Thus, based on the experiments of Hayes et al.^9^, it cannot be determined whether the re-appearance of the learning context only caused a change in the sense of familiarity of the objects or increased the discrimination of similar items. Also, we cannot make a clear conclusion whether the subject had access to the contextual information during the recognition test.

### The difference between target identification and mnemonic discrimination

A possible solution to the contradiction in the results revealing the effect of context on recognition is to consider that identifying a target and discriminating a similar lure may involve different computational and neural processes^10^. Capturing and recalling unique memories that can be placed in space and time are key functions of episodic memory^11,12^. Computational models of hippocampal functioning have uncovered the mechanism by which unique, distinguishable memory representations are generated on the basis of many similar events^13–15^. This computational mechanism of the hippocampus, called pattern separation, is suggested to be responsible for the reduction of interference effects between overlapping memory representations^10,16,17^. At a neural level, as a result of pattern separation, activities of brain circuits become distinct for stimuli that share similar features^16,18,19^. At a behavioral level, individuals become able to identify (and correctly reject) a stimulus that is similar to a previously perceived item—a process often referred to as mnemonic discrimination^20–22^. Some computational models of the hippocampus suggest that before the correct rejection of a similar stimulus, one needs to recall the previously perceived item to detect differences between them^15,20^, a process called the “recall-to-reject” strategy^23,24^. Strongly related to these concepts, another hippocampal computational mechanism (pattern completion), refers to the processes when a memory becomes accessible following the presentation of a partial (degraded) cue^10,16,17^.

The behavioral manifestation of putative neural mechanisms enabling pattern separation and completion is typically assessed by different types of item recognition memory tasks. In the past few years, the Mnemonic Similarity Task (MST)^21^ became a benchmark test to examine hippocampal integrity in ageing and in various disorders^22,25,26^. In this task, participants are presented with images of everyday objects. On the test, participants are shown previously seen target items and completely new foils. Importantly, critical lure items are also presented that are visually similar to the target items. While “old” responses given to the lure items are suggested to reflect pattern completion, “similar” responses given to the lures are assumed to be associated with pattern separation.

Crucially, the two concepts (pattern separation and mnemonic discrimination) are associated with descriptions of different levels in explanation. While pattern separation is a computational process, mnemonic discrimination reflects behavioral performance in a memory task in which interference resolution is needed for the correct discrimination between the target items and their corresponding lures (for overviews, see^10,21^). In other words, naturally, there is no one-to-one mapping between the computation and behavior. However, a causal relationship can be assumed between them, as for the correct discrimination between a target item and its visually similar lure, interference resolution is needed, and pattern separation is the process that is responsible for the orthogonalization of overlapping representations at the computational level.

The strong relationship between pattern separation and mnemonic discrimination is also confirmed by previous neuroimaging studies. There is a long line of studies suggesting that the dentate gyrus and the CA3 subregion of the hippocampus perform pattern separation (e.g.^27^). Accordingly, the activations of these areas were also shown to be associated with mnemonic discrimination in the MST (e.g.^18,28^; for an overview, see^10^).

### Study objectives

**Figure 1:**
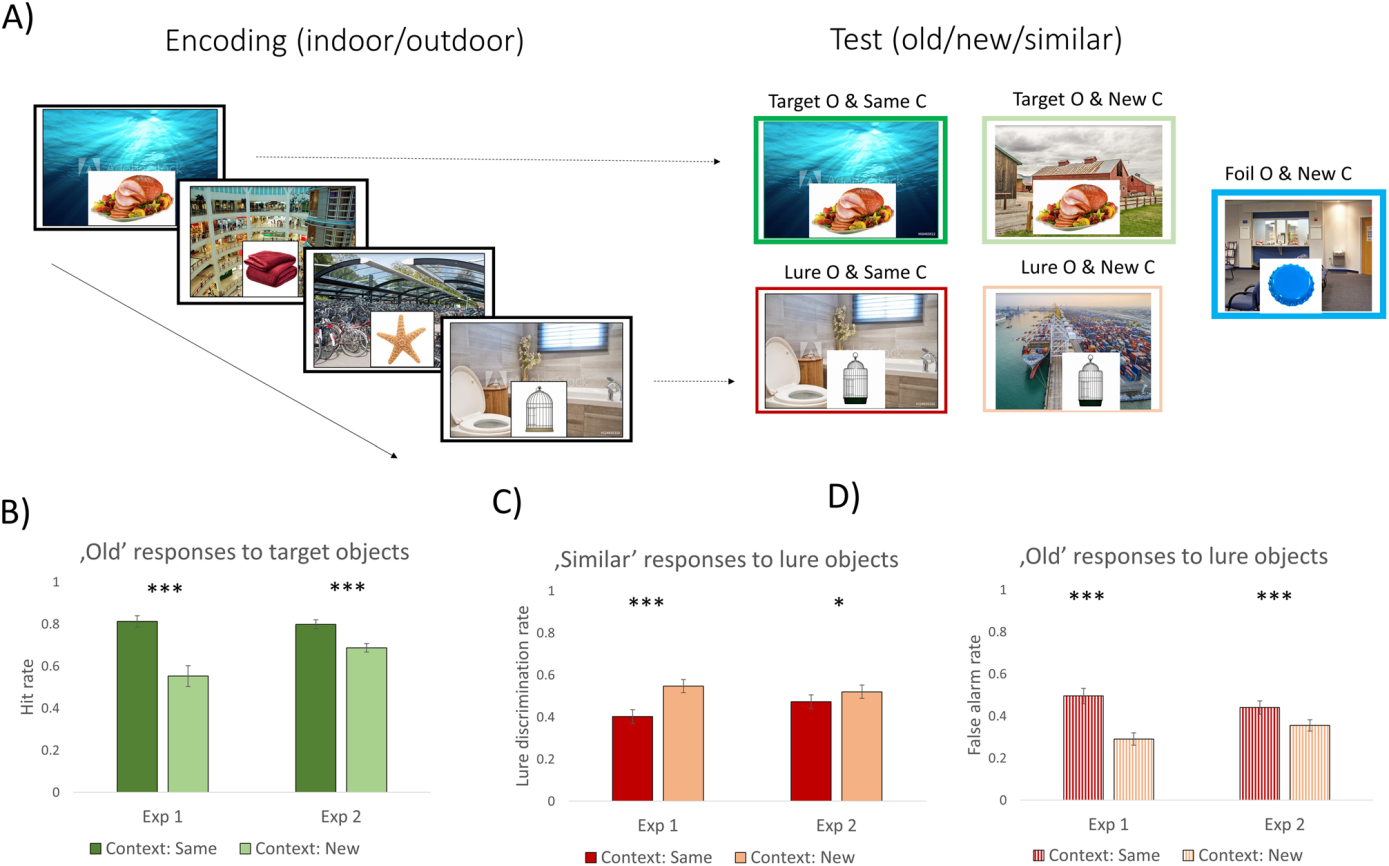
The procedure (**A**) and the results (‘**B**’, ‘**C**’, ‘**D**’) of Experiment 1 and Experiment 2. Note(s). In both experiments (‘**A**’), participants made indoor/outdoor judgements on photographs of everyday objects presented on a background of scene images. They were specifically instructed to judge the objects only. Later, in a surprise recognition memory task, these original target objects were presented together with similar lure objects and completely new foils. Each target and lure object was presented either on the same or on a new background context. Participants were instructed to make an “old”/“similar”/“new” decision. In Experiment 2, participants were explicitly instructed to make their judgements based on the object, whereas in Experiment 1 no such instruction was given. (Experiment 1: N = 28; Experiment 2: N = 40; O: Object; C: Context.) We investigated whether context type affects the ratio of “old” responses given to the target objects (‘**B**’) as well as the ratios of “similar” (‘**C**’) and “old” (‘**D**’) responses given to the lure objects. Error bars represent the standard error of the mean. *p < 0.05; ***p < 0.001.

Although the use of contextual information is one of the most important features of episodic memory, human data on the role of contextual details in episodic recognition memory and mnemonic discrimination is sparse and highly controversial. To test these questions, we conducted two experiments. Both experiments started with an incidental encoding phase, where participants first saw pictures of everyday objects overlaid on a background scene picture (see Fig. 1A). Their task was to make an indoor/outdoor decision about the object. That is, no memory instruction was given and so the encoding of the object-scene pairs was incidental. Crucially, the task was always associated with the object and so the scene picture served only as a background context. After this, memory was tested on a surprise recognition memory test, in which some of the objects from the encoding phase were presented again (targets). Additionally, we presented new objects that were similar but not identical to the previously seen objects (lures) as well as new objects that were not similar to the target objects (foils). The task of the participants was to discriminate between these object types. Our experimental manipulations in each experiment targeted how the background context influences mnemonic discrimination during the test phase. In both experiments, we predicted that manipulating the non-attended background context will affect how participants discriminate between target, lure, and foil objects.

In Experiment 1, participants were asked to make “old”/“similar”/“new” decisions about the picture they saw. No specific instruction was given about whether participants have to focus their attention on the object in the bottom center or on the whole display, together with the background. To ensure that participants make their memory judgments focusing on the object only, in Experiment 2, participants were explicitly instructed to focus on the object only and not on the whole picture together with the background context.

## Results

The results are presented in Fig. 1B–D. Our results show that irrespective of the specific instruction, presenting the target objects on the previously studied context is associated with better target identification performance in both Experiment 1, *t*(27) = 5.500, *p* < 0.001, *d* = 1.054, and Experiment 2, *t*(39) = 4.601, *p* < 0.001, *d* = 0.615 (see Fig. 1B). In contrast, lure discrimination was impaired when the lure objects were presented on the same background scene which was previously paired with the original version of the object. Such lure items were less frequently labelled similar in both Experiment 1, *t*(27) = 5.212, *p* < 0.001, *d* = 0.464, and Experiment 2, *Z* = 2.386, *p* = 0.017, *r* = 0.267 (see Fig. 1C). In contrast, such lure objects were more often recognized falsely as old in both Experiment 1, *t*(27) = 5.066, *p* < 0.001, *d* = 0.947, and Experiment 2, *Z* = 3.347, *p* < 0.001, *r* = 0.374 (see Fig. 1D). That is, the unattended background context influenced both recognition hits and lure discrimination during the test phase. We asked participants to make confidence judgments to make sure that they attended to the stimuli and were not just guessing when they made the “old”/“similar”/“new” decisions. Based on the results, mean confidence ratings were higher than 5 for the correct responses (“old” for targets and “similar” for lures), suggesting that participants were not just guessing on the recognition memory test (for the descriptive statistics, see Tables S1 and S2).

## Discussion

Here we have demonstrated that irrelevant background information is embedded in episodic memories in detail and its reappearance impairs mnemonic discrimination functions. In two experiments, using two types of testing instructions, we showed that the reappearance of an unattended background context after incidental learning increased recognition hit performance. However, the background context not only had a positive effect on recognition: when it appeared together with similar lure elements, it also reduced mnemonic discrimination and increased the rate of false recognition of the lure items. Therefore, presumably, the reappearance of the background context led to a pattern completion bias, that is, participants retrieved the original target item (pattern completion), instead of labelling the lure items as similar (pattern separation). Our results suggest that the background context is encoded in detail along with the target information, and on a recognition test, subjects use a detail-rich context representation to identify the target. At the same time, mnemonic discrimination performance, which reflects the pattern separation process, appears to be modulated only at the level of shallow familiarity by the background context. This result is consistent with the recently reported results of Szőllősi et al.^29^ showing that mnemonic discrimination is primarily modulated by familiarity-based decisions.

Altogether these results demonstrate that during episodic recognition background information modulates processes leading to correct and false recognition, respectively. The reappearance of the background context of the encoding situation increases the familiarity of the target elements and thus aids in the recognition identification, however also raising the familiarity of similar elements, reducing their correct discrimination. These results are consistent with theories that assume that random contextual information present at the original event during incidental encoding of individual memories is also part of a memory representation, and that this contextual information is mobilized by the hippocampal complex during episodic recognition^30–36^. The assumptions that irrelevant contextual information is strongly associated with episodic memories and that the hippocampal complex has a crucial role in forming this association are also consistent with the functional magnetic resonance imaging (fMRI) results of Hayes et al.^9^. They found that parahippocampal complex (PHC) activation during incidental encoding of objects was associated with a subsequent context-change effect on correct recognition rates. Interestingly, they found greater activity in the bilateral PHC at retrieval when the object was presented on a blank white background (as opposed to its original detailed visual background at incidental encoding), a result suggesting that the PHC may reinstate visual context to mediate successful episodic recognition. Hayes et al.^9^ interpreted their findings to be consistent with the Multiple Trace Theory (MTT)^30–34^. The MTT states that the hippocampal complex is involved in the storage and retrieval of contextual information, specifically, the authors assume that the PHC together with the hippocampal complex is involved in both incidental encoding and the retrieval of scene contextual information^9^. However, the results of our study draw attention to the fact that, unlike the results of Hayes et al.^9^, the incidentally encoded background context has more than positive effects on recognition performance. We suggest that the re-appearance of the learning context during the recognition test raises the level of familiarity of similar lure elements and thus degrades memory discrimination performance.

One important theoretical framework in the false recognition literature is the fuzzy-trace theory (FTT^37,38^). The most important assumption of the FTT is that recognition decisions can be made on the basis of operations on two independent representations, which are the verbatim trace (surface form) and the gist trace (meaning content). While the former contains elements of the encoded experience, including contextual cues, the latter contains the semantics (meaning, relations, patterns) of the event. Activation of the verbatim traces promotes the vivid and recollective contextual re-experience of events, while retrieval of gist traces is associated with familiarity-based memory decisions^37^.

According to the FTT, the “similar” decision given to the lures in the MST and the related test question require a verbatim answer, because gist-based information is not sufficient for subtle memory discrimination between semantically identical lure and target items. However, based on the FTT, two conclusions can be drawn from our data.

First, it might be the case that the re-appearance of an unattended and task-irrelevant visual background scene shifts performance toward a gist-like decision, thereby increasing false memory and decreasing memory discrimination. This is because target and lure objects are not only visually similar but they also have the same background, and so the verbatim representation does not allow for discrimination anymore: it is largely the same for the target and the lure object, and so the participant is forced to make a gist- (meaning-) based decision. Consequently, the number of “old” responses to the lure objects will increase and the number of “similar” responses to the lure objects will decrease. In other words, the false memory rate of lures will increase, and lure discrimination will decrease.

Second, the unattended and irrelevant background might be included in the verbatim representation together with the object. During the test, due to the reappearance of the background scene together with a lure object, the verbatim representation of the target and lure will overlap to a high degree to increase the false alarm and reduce lure discrimination even in case of verbatim-based response. This explanation seems to be probable based on a recent study of Nieznański and Tkaczyk^39^. Motivated by the FTT, they investigated the role of background context reinstatement for the emergence of false recognition. Although this study differed from our study in a number of important aspects, making it difficult to make direct comparisons, it nevertheless appears to provide evidence convergent with our data. In this study verbal items appeared on unique background images during encoding, and were then presented during the recognition test either on the original background or on the background of another item (context switch) or without a background. The most significant differences from our paradigm were as follows: unlike our study, they used intentional learning where participants were instructed to establish a mental association between background and item. During the recognition test, the lures were semantic and the context manipulation was between subjects. Despite all these significant differences, it is important that this study concluded that the re-appearance of background context increased access to verbatim representation.

Whichever version of the explanation is true, in both cases the FTT concludes that unique and unattended background is encoded in the verbatim representation of the incidentally acquired object, and participants could access this item + context ensemble representation^40^ during the test. Consequently, it seems to be a tentative assumption that verbatim ensemble representations influence participants’ decision-making mechanisms for mnemonic discrimination.”

Our study has important limitations. First, in research on the effects of background context, a control condition without background context is often used, to disentangle effects specifically related to the presence of a background context. Second, our design is unbalanced with respect to the context manipulation in association with foils: that is, foils in the test were always presented on new background scenes, and never with previously seen background context. These two features of the design were chosen to keep the amount of to-be-presented information relatively low and so to avoid floor effects during the test and also to ensure that there is minimum interference between our stimuli (e.g., with increasing set size, it becomes increasingly difficult to ensure that the background contexts are not related semantically). Nevertheless, we suggest that these design choices are not threatening the reliability and validity of our findings, as they are independent from the main focus of our inquiry: the manipulation of background context familiarity during target detection and lure discrimination.

It is important for future research to explore the relationship between individual differences in the processing of unattended and irrelevant contextual information and changes in the pattern separation process in older age. Another important direction might be to manipulate variables affecting the encoding and consolidation of the information: e.g. by using intentional encoding or a long delay between encoding and test. Varying the test format might also lead to further insight: by asking for a Remember/Know decision^41^ during the test, the respective roles of familiarity and recollection could be explored. Furthermore, inserting a test condition, where target items are shown without a background scene might enable us to better specify the role of unattended background information on mnemonic discrimination. Finally, building on the literature on the neural background of lure discrimination, it would be interesting to see how manipulating the background context might affect neural pattern separation carried out by the hippocampus.

In sum, the presented findings support the idea that unattended, irrelevant context information has a negative effect on the pattern separation process. These results are particularly noteworthy given that behavioral indicators that reflect the pattern separation process (e.g., lure discrimination index) are sensitive predictors of mild cognitive impairment (MCI) and dementia^22,26^. Future research is important to explore the relationship between individual differences in the processing of unattended and irrelevant contextual information and changes in the pattern separation process in older age.

## Materials and methods

### Participants

Participants were undergraduate students. They received either course credit or money for participation. All participants gave written informed consent. They had no history of psychiatric or neurological disorders and had normal or corrected-to-normal vision. In Experiment 1 two participants were excluded from the sample for not giving any correct response in one of the conditions. Therefore, we analyzed the data of 28 participants in Experiment 1 (19 females; age range: 19–26 years, *M* = 22.7, *SD* = 1.9) and the data of 40 participants in Experiment 2 (26 females; age range: 18–30 years, *M* = 22.1, *SD* = 2.4).

The study was approved by the Hungarian United Ethical Review Committee for Research in Psychology. The experiments were carried out in accordance with the Code of Ethics of the World Medical Association (Declaration of Helsinki) for experiments involving humans.

### Stimuli

Stimuli were color photographs of everyday objects and color photographs of scenes. Each object was presented on the background of a unique scene at encoding. We refer to these background scenes as “contexts” in the following. The background scenes were either taken from a commercial stock-photo database or selected from freely available internet data sources. Object images were adapted from Stark and colleagues (https://github.com/celstark/MST). The similarity level of the lure objects in this version of the task was determined by the rates of false alarm judgements given to the specific lures on a memory test performed by an independent group of participants^28^. The objects and their unique contexts were randomly assigned into pairs. The object and the corresponding context image were semantically unrelated. The objects were seen at the bottom center of the computer screen. While the objects’ presentation size was 5.5° visual angles in height, the contexts’ presentation size was 17.3° visual angles in height.

### Experimental design and procedure

We conducted two experiments. Both experiments consisted of two main phases, an incidental encoding phase and a recognition memory test. The experimental procedure is illustrated in Fig. 1A. The two experiments’ differed only in the instruction presented before the recognition test phase.

In both experiments 60 object-context pairs were presented at encoding (4 s/stimulus pair, pre-stimulus interval [PSI] = 1 s). Participants were instructed to make an indoor/outdoor decision about the objects. The response buttons were V (indoor) and N (outdoor) on a standard keyboard of the computer.

In the recognition test phase participants were presented with 90 object-context pairs. We used a 3 × 2 (Object × Context) experimental design. There were three object types (with 30 images in each condition): targets, lures, and foils. Target images were exact repetitions of ones presented at encoding, whereas foils were completely new images. Lures were visually similar images to ones presented in the encoding phase. These objects were presented on either the same or a new background context in the recognition memory task. In other words, half of the target objects appeared either on the same background as in the encoding phase of the task (same condition) or on a completely new background which was not presented at all before (new condition). Lure items were also presented either on the same context (i.e., on the same background as the corresponding original image was presented at encoding) or on a new context. Additionally, each foil was presented on a new, unique context.

Participants’ task was to make “old”/“similar”/“new” decisions. Specifically, they were instructed to decide whether the image is a target (“old”), a lure (“similar”), or a foil (“new”) item. The response buttons were F, H, and K, respectively. Participants had 4 s to respond (PSI = 0.5 s). Each “old”/“similar”/“new” decision was followed by a secondary recognition confidence judgment where participants had 5 s to respond. Specifically, they were required to rate after each response how confident they were that they had made the right decision. The scale ranged between 1 = “Not at all sure” and 6 = “Very sure”. There was a 90-s practice phase before the recognition test in both experiments while participants were presented with the labels of the confidence scale (e.g., “Not at all sure”) and were required to press the corresponding response button.

The two experiments differed only in the instruction presented immediately before the recognition test. In Experiment 1 we used the standard instruction of the MST. Specifically, participants were asked to make an “old”/“similar”/“new” about the picture they saw. To ensure that participants make their memory judgments focusing on the object only, in Experiment 2, participants were explicitly instructed to focus on the object only and not on the whole picture together with the background context.

### Data analysis

We analyzed the hit rate of target objects (the ratio of “old” responses for targets), as well as lure discrimination and false recognition of lures (ratio of “similar” and “old” responses given to the lure images, respectively). To investigate the effect of unattended background context on these measures, we conducted pairwise comparisons of the same and new context conditions in both Experiment 1 and Experiment 2. For normally distributed data, we conducted two-tailed paired-samples t-tests. In cases where the data did not follow normal distribution, we used Wilcoxon signed ranks tests for comparing the same and different context conditions.

Note that during our analysis of successful lure discrimination, we diverted from the standard practice in MST, which suggest to correct for the general tendency of responding with “similar” by subtracting the number of “similar” responses to foils from the number of “similar” responses to lures. In our case, however, such correction would have not changed the pattern of our results, as the same number should have been subtracted from the two experimental conditions.

## Supplementary Information

Supplementary Information

## Data Availability

Datasets related to this article are available at Open Science Framework (https://osf.io/g7qe3/).

## References

[1] Baddeley AD (1999). Essentials of Human Memory.

[2] Locke, J. *An Essay Concerning Human Understanding* (University Press, 1817/1960).

[3] Smith SM, Glenberg A, Bjork RA (1978). Environmental context and human memory. Mem. Cogn..

[4] Smith SM, Vela E (2001). Environmental context-dependent memory: A review and meta-analysis. Psychon. Bull. Rev..

[5] Isarida T, Isarida TK (2014). Environmental Context-Dependent Memory.

[6] Godden D, Baddeley A (1980). When does context influence recognition memory?. Br. J. Psychol..

[7] Baddeley AD, Woodhead M (1982). Depth of processing, context, and face recognition. Can. J. Psychol..

[8] Watkins MJ, Ho E, Tulving E (1976). Context effects in recognition memory for faces. J. Verb. Learn. Verb. Behav..

[9] Hayes SM, Nadel L, Ryan L (2007). The effect of scene context on episodic object recognition: Parahippocampal cortex mediates memory encoding and retrieval success. Hippocampus.

[10] Yassa MA, Stark CE (2011). Pattern separation in the hippocampus. Trends Neurosci..

[11] Conway MA (2009). Episodic memories. Neuropsychologia.

[12] Tulving E (1985). How many memory systems are there?. Am. Psychol..

[13] Marr D, Willshaw D, McNaughton B (1991). Simple Memory: A Theory for Archicortex.

[14] McClelland JL, McNaughton BL, O'Reilly RC (1995). Why there are complementary learning systems in the hippocampus and neocortex: Insights from the successes and failures of connectionist models of learning and memory. Psychol. Rev..

[15] Norman KA, O'Reilly RC (2003). Modeling hippocampal and neocortical contributions to recognition memory: A complementary-learning-systems approach. Psychol. Rev..

[16] Hunsaker MR, Kesner RP (2013). The operation of pattern separation and pattern completion processes associated with different attributes or domains of memory. Neurosci. Biobehav. Rev..

[17] Rolls E (2013). The mechanisms for pattern completion and pattern separation in the hippocampus. Front. Syst. Neurosci..

[18] Bakker A, Kirwan CB, Miller M, Stark CE (2008). Pattern separation in the human hippocampal CA3 and dentate gyrus. Science.

[19] Gilbert PE, Kesner RP (2006). The role of the dorsal CA3 hippocampal subregion in spatial working memory and pattern separation. Behav. Brain Res..

[20] Kirwan CB, Stark CE (2007). Overcoming interference: An fMRI investigation of pattern separation in the medial temporal lobe. Learn. Mem..

[21] Stark SM, Kirwan CB, Stark CE (2019). Mnemonic similarity task: A tool for assessing hippocampal integrity. Trends Cogn. Neurosci..

[22] Stark SM, Yassa MA, Lacy JW, Stark CE (2013). A task to assess behavioral pattern separation (BPS) in humans: Data from healthy aging and mild cognitive impairment. Neuropsychologia.

[23] Rotello CM, Heit E (1999). Two-process models of recognition memory: Evidence for recall-to-reject?. J. Mem. Lang..

[24] Rotello CM, Macmillan NA, Van Tassel G (2000). Recall-to-reject in recognition: Evidence from ROC curves. J. Mem. Lang..

[25] Ally BA, Hussey EP, Ko PC, Molitor RJ (2013). Pattern separation and pattern completion in Alzheimer's disease: Evidence of rapid forgetting in amnestic mild cognitive impairment. Hippocampus.

[26] Stark SM, Stark CE (2017). Age-related deficits in the mnemonic similarity task for objects and scenes. Behav. Brain Res..

[27] Leutgeb JK, Leutgeb S, Moser MB, Moser EI (2007). Pattern separation in the dentate gyrus and CA3 of the hippocampus. Science.

[28] Lacy JW, Yassa MA, Stark SM, Muftuler LT, Stark CE (2011). Distinct pattern separation related transfer functions in human CA3/dentate and CA1 revealed using high-resolution fMRI and variable mnemonic similarity. Learn. Mem..

[29] Szőllősi Á, Bencze D, Racsmány M (2020). Behavioural pattern separation is strongly associated with familiarity-based decisions. Memory.

[30] Moscovitch M, Nadel L (1998). Consolidation and the hippocampal complex revisited: In defense of the multiple-trace model. Curr. Opin. Neuribiol..

[31] Nadel L, Moscovitch M (1997). Memory consolidation, retrograde amnesia and the hippocampal complex. Curr. Opin. Neuribiol..

[32] Nadel L, Moscovitch M (1998). Hippocampal contributions to cortical plasticity. Neuropharmacology.

[33] Nadel L, Ryan L, Hayes SM, Gilboa A, Moscovitch M (2003). The role of the hippocampal complex in long-term episodic memory. Intern. Cong. Ser..

[34] Nadel L, Samsonovich A, Ryan L, Moscovitch M (2000). Multiple trace theory of human memory: Computational, neuroimaging, and neuropsychological results. Hippocampus.

[35] Norman KA (2010). How hippocampus and cortex contribute to recognition memory: Revisiting the complementary learning systems model. Hippocampus.

[36] Yonelinas AP, Aly M, Wang WC, Koen JD (2010). Recollection and familiarity: Examining controversial assumptions and new directions. Hippocampus.

[37] Brainerd CJ, Reyna VF (2002). Fuzzy-trace theory and false memory. Curr. Dir. Psychol. Sci..

[38] Reyna VF, Brainerd CJ (1995). Fuzzy-trace theory: An interim synthesis. Learn. Individ. Diff..

[39] Nieznański M, Tkaczyk D (2017). Effects of pictorial context reinstatement on correct and false recognition memory: Insights from the simplified conjoint recognition paradigm. J. Cogn. Psychol..

[40] Murnane K, Phelps MP, Malmberg K (1999). Context-dependent recognition memory: The ICE theory. J. Exp. Psychol. Gen..

[41] Tulving E (1985). Memory and consciousness. Can. Psychol..

