# Supplementary material for: Irrelevant background context decreases mnemonic discrimination and increases false memory
Source: Sci Rep. 2021 Mar 18;11:6204. doi: 10.1038/s41598-021-85627-2 (PMC7973510; doi:10.1038/s41598-021-85627-2)
Supplement: Supplementary file 1 — Supplementary Information [file 41598_2021_85627_MOESM1_ESM.docx]

**Supporting Online Material**

**Irrelevant background context decreases mnemonic discrimination and increases false memory**

Mihály Racsmány^1,2 *^, Dorottya Bencze^1,2^, Péter Pajkossy^1,2^, Ágnes Szőllősi^1,2^, and Miklós Marián^1^

^1^ *Department of Cognitive Science, Budapest University of Technology and Economics, Hungary*

^2^ *Institute of Cognitive Neuroscience and Psychology, Research Centre for Natural Sciences, Hungary*

Supporting Tables

**Table S1**

*Response rates and mean confidence ratings for “old”, “similar”, and “new” responses given to the target, lure, and foil items presented on the original (same) or new backgrounds (Experiment 1).*

| **Stimulus type** | **Response type** | **Response rate** | | **Mean confidence rating** | |
| --- | --- | --- | --- | --- | --- |
|  |  | **Same context** | **New context** | **Same context** | **New context** |
| **Targets** | **Old** | 0.81 (0.03) | 0.55 (0.05) | 5.75 (0.08) | 5.38 (0.14) |
|  | **Similar** | 0.15 (0.03) | 0.37 (0.04) | 4.21 (0.20) | 4.70 (0.15) |
|  | **New** | 0.02 (0.01) | 0.04 (0.01) | 3.36 (0.71) | 4.25 (0.53) |
|  |  |  |  |  |  |
| **Lures** | **Old** | 0.50 (0.04) | 0.29 (0.03) | 5.66 (0.09) | 5.17 (0.18) |
|  | **Similar** | 0.40 (0.03) | 0.55 (0.03) | 5.20 (0.14) | 4.88 (0.17) |
|  | **New** | 0.06 (0.02) | 0.11 (0.02) | 4.63 (0.43) | 4.57 (0.34) |
|  |  |  |  |  |  |
| **Foils** | **Old** |  | 0.02 (0.01) |  | 3.96 (0.43) |
|  | **Similar** |  | 0.07 (0.01) |  | 3.76 (0.33) |
|  | **New** |  | 0.88 (0.02) |  | 5.55 (0.12) |
|  |  |  |  |  |  |

*Note(s).* Standard errors of the means are shown in parentheses.

**Table S2**

*Response rates and mean confidence ratings for “old”, “similar”, and “new” responses given to the target, lure, and foil items presented on the original (same) or new backgrounds (Experiment 2).*

| **Stimulus type** | **Response type** | **Response rate** | | **Mean confidence rating** | |
| --- | --- | --- | --- | --- | --- |
|  |  | **Same context** | **New context** | **Same context** | **New context** |
| **Targets** | **Old** | 0.80 (0.02) | 0.69 (0.02) | 5.74 (0.08) | 5.65 (0.11) |
|  | **Similar** | 0.14 (0.02) | 0.23 (0.02) | 4.63 (0.16) | 4.40 (0.13) |
|  | **New** | 0.04 (0.01) | 0.06 (0.01) | 4.19 (0.41) | 4.58 (0.26) |
|  |  |  |  |  |  |
| **Lures** | **Old** | 0.44 (0.03) | 0.36 (0.03) | 5.55 (0.11) | 5.46 (0.13) |
|  | **Similar** | 0.47 (0.03) | 0.52 (0.03) | 4.99 (0.13) | 5.04 (0.12) |
|  | **New** | 0.06 (0.01) | 0.09 (0.02) | 4.43 (0.34) | 5.02 (0.22) |
|  |  |  |  |  |  |
| **Foils** | **Old** |  | 0.03 (0.01) |  | 3.80 (0.31) |
|  | **Similar** |  | 0.11 (0.02) |  | 4.13 (0.20) |
|  | **New** |  | 0.85 (0.02) |  | 5.41 (0.11) |
|  |  |  |  |  |  |

*Note(s).* Standard errors of the means are shown in parentheses.
